# The importance of α-CT and Salt bridges in the Formation of Insulin and its Receptor Complex by Computational Simulation

**Published:** 2018

**Authors:** Marzieh Dehghan-Shasaltaneh, Hossein Lanjanian, Gholam Hossein Riazi, Ali Masoudi-Nejad

**Affiliations:** a *Laboratory of Systems Biology and Bioinformatics (LBB), Institute of Biochemistry and Biophysics, University of Tehran, Tehran, Iran. *; b *Laboratory of Neuro-organic Chemistry, Institute of Biochemistry and Biophysics (IBB), University of Tehran, Tehran, Iran.*

**Keywords:** Insulin, Insulin receptor, HADDOCK, Conformational changes, Salt bridges

## Abstract

Insulin hormone is an important part of the endocrine system. It contains two polypeptide chains and plays a pivotal role in regulating carbohydrate metabolism. Insulin receptors (IR) located on cell surface interacts with insulin to control the intake of glucose. Although several studies have tried to clarify the interaction between insulin and its receptor, the mechanism of this interaction remains elusive because of the receptor’s structural complexity and structural changes during the interaction. In this work, we tried to fractionate the interactions. Therefore, sequential docking method utilization of HADDOCK was used to achieve the mentioned goal, so the following processes were done: the first, two pdb files of IR i.e., 3LOH and 3W11 were concatenated using modeller. The second, flexible regions of IR were predicted by HingeProt. Output files resulting from HingeProt were uploaded into HADDOCK. Our results predict new salt bridges in the complex and emphasize on the role of salt bridges to maintain an inverted V structure of IR. Having an inverted V structure leads to activate intracellular signaling pathway. In addition to presence salt bridges to form a convenient structure of IR, the importance of α-chain of carboxyl terminal (α-CT) to interact with insulin was surveyed and also foretokened new insulin/IR contacts, particularly at site 2 (rigid parts 2 and 3). Finally, several conformational changes in residues Asn711-Val715 of α-CT were occurred, we suggest that α-CT is a suitable situation relative to insulin due to these conformational alterations.

## Introduction

Diabetes mellitus is one of the most significant diseases, resulting from severe metabolic imbalances. Regarding this issue, researchers pay attention to pancreas which involves in creating oxidative stress (1). Insufficient levels of insulin or inhibition of antioxidants enzymes such as superoxide dismutase in diabetic patients cause oxidative stress and may damage or kill cells (2).

Insulin is a peptide hormone composed of 51 amino acids with a molecular weight of 5808 Da. It contains two chains: a 21 residue A-chain and a 30 residue B-chain joined by two disulfide bonds. Insulin plays a significant role in regulating carbohydrate and fat metabolism in animal cells. By initiating and promoting biosynthesis and storage of carbohydrates, lipids and proteins, insulin inhibits their degradation and is released back into the blood circulation (3).

Insulin is produced by beta cells in the islets of Langerhans. The Hexamer form of insulin often incorporates zinc which makes it more stable, but it is released into the blood stream as a zinc free monomer. After the release, insulin binds to its receptor on target cells (4-6).

Insulin receptor (IR) is a membrane protein that starts a cascade of events in the presence of insulin (5, 7). IR is a disulfide-bound (αβ)_2 _homodimer protein composed of two regions including two extracellular subunits with 135 KD R-subunits and two 95 KD transmembrane β- subunits (5, 8). 

The extracellular portion of each αβ subunit, i.e., the ectodomain, contains six domains including a leucine-rich repeat domain, L1 ectodomain, a cycteine-rich domain, CR, a second leucine-rich repeat domain L2 and three fibronectin type-III domains: FnIII-1, FnIII-2, and FnIII-3. There is also an insert domain, ID with FnIII-2. The α-chain component of the ID is terminated by a segment known as αCT (α-chain) (α-chain of carboxyl terminal) which consists of residues 704-719 (4, 8, 9).

In the presence of insulin, the IR ectodomain is folded into an inverted ‘V’ shape. Each leg of the inverted ‘V’ consists of the L1-CR-L2 module in an extended conformation juxtaposed against an extended linear arrangement of the three fibronectine two-fold symmetry. At the apex of the inverted ‘V’, the L2 domain of each monomer interacts with the FnIII-1 domain of the another monomer, while at the midpoint of the legs, the L1 domain of each monomer is in contact with the FnIII-2 domain of the secondary monomer (7, 10, 11). In the literature, to distinguish the IR monomers, one of them is denoted by “ ′ “.Unfortunately, the total crystal structure of IR is not available. Michael C. Lawrence and co-workers have released several crystal structures of the insulin receptor from 2006 to 2013 (such as Protein Data Bank accession code 2DTG, 3LOH and 3W11) which were derived from X-ray diffraction data (8). 

In the current model of insulin-receptor complex, the binding is mediated by two adjoining structural regions: a low affinity site1 and a high affinity site2 (9). 

Extensive studies on mutant receptors have established that site 1 is composed of two distinct regions: i) the center of three β-sheets of L1 domain and the central modules in the CR region of the first monomer, ii) the last 16 residues of the α-CT of the other monomer. Site 2 is possibly formed by loops at the junction of the FnIII-1 and FnIII-2 domains of the opposite monomer (12).

There are two pockets found on the insulin receptor molecule. One formed by L1-CR-L2 (site 1) and (F1-F2-F3)′ (site 2′) domains and the other one formed by (L1-CR-L2)′ (site 1′) and (F1-F2-F3) (site 2) domains. 

As mentioned above, there is just one pocket on each leg of IR dimer. The current belief for insulin/receptor binding mechanism is as follows: one insulin molecule docks at the accessible pocket and causes two monomers to close up and to open up the opposite site, making it accessible to other insulin molecules. This situation leads to accelerate dissociation of the first insulin at the site 1/site 2′ (first pocket) (12).

Although several studies have tried to clarify the interaction between insulin and its receptor, the mechanism of this interaction remains elusive because of the receptor’s structural complexity and structural changes during the interaction. In this work, we tried to fractionate the interactions. Therefore, sequential docking method utilization of HADDOCK was used to achieve the mentioned goal.

## Experimental

*Input files*

Input coordinates for human insulin were obtained from the RCSB Data Bank (PDB code 3INC). The IR’s ectodomain which was used in this study contains domains L1, CR, L2, F1, F2 and F3, but lacks a part of the insert domain (residues 656-692 and 711-754) including the CT peptide. Input coordinates for the human IR were obtained from 3.8 Å and 3.9 Å resolution X-ray crystallographic data which include PDB code 3LOH and 3W11, respectively (9). After combination two pdb files of IR using modeler, several servers were used to evaluate the final model such as PROCHECK (13), QMEAN (14), and RAMPAGE (15). 

Because of inability of crystallography methods to determine a complete structure of α-CT of IR, 3LOH and 3W11 pdb files were concatenated using modeler 9.12 to create a pdb file contains residues 693-715. A default template has not been determined for residues 716-719 (8).

*Determination of the hinge regions*

The hinge prediction server, Hinge Prot, was used to predict hinge regions of flexible monomers. HingeProt annotates rigid parts and possible hinges of the supplied protein based on two Elastic Network Models (GNM) (16) and Anisotropic Network Model (ANM) (17). 

In this work, each monomer of IR was dissected by HingeProt server and two different sets of output files were provided, i.e., rigid parts, and Hinge residues (Table 1). The detailed procedure is described in the result section. HingeProt cluster the fragments into structurally compact rigid and score the obtained prediction defined based on the reference (18).

*Docking protocol of HADDOCK*

In order to simulate insulin/IR’s model, we focused on our data-driven docking approach, i.e., HADDOCK using sequential docking method (19-21). HADDOCK is an information- driven flexible docking approach for the modeling of bimolecular complexes. It can account for conformational changes occurring upon binding using explicit flexible during the molecular dynamics refinement. Since molecular dynamics isn’t really suited for docking purpose because sampling in docking would be extremely computational expensive, so HADDOCK could be used to refine model (20).

**Figure 1:**
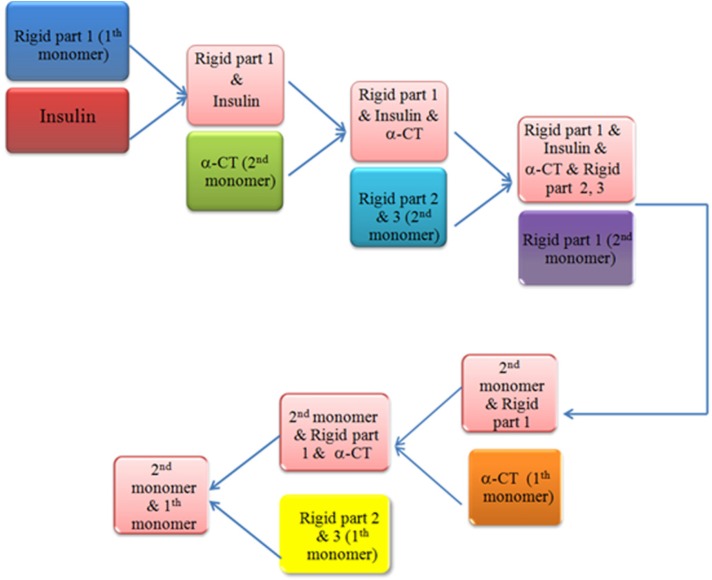
Work-flow of sequential docking process. After disruption our receptor by HingeProt, each part was uploaded in HADDOCK and the best solution resulted from each process was used for the next step. Rigid part 1: L1 domain of insulin receptor, Rigid part 2 & 3: FnIII-1 and FnIII-2 of insulin receptor

The docking protocol comprise of three steps, a rigid-body energy minimization, a semi-flexible refinement in torsion angle space and a final refinement in explicit solvent (20). Based on Michael’s literature (22) we decided to choose the order of components involving in the interaction. Insulin bonded to all components of IR according to the following order: L1 domain (first monomer), α-CT, FIII-1, FIII-2, FIII-3 and L1 domains (second monomer), α-CT and FIII-1, FIII-2, FIII-3 domains of the first monomer. We also evaluated simultaneously docking method as an alternative approach, but the result has a lower compatible with experimental data that confirms the order of components (data not shown). Work flows of the sequential docking method have been shown in Figure 1.

*Sequential docking method*

After uploading pdb file of IR resulting from HingeProt step by step and insulin pdb file, several parameters were changed in HADDOCK according to the following steps; ambiguous restraints of guru-interface were determined based on the active and passive residues resulting from the literature (8, 23) using HADDOCK (http://haddock.science.uu.nl/services/GenTBL/). Unambiguous restraints manually created a restraint between salt bridges and in the hinge regions of IR homodimer. “Center of mass restraints” between domains were turned off and AIRs (Randomly exclude a fraction of the ambiguous restraints) was turned on. “The number of structures” was increased to 10000, 400, and 400 for it0, it1 and water, respectively. The rest of the parameters were left to their default values (24). 

To analysis hydrophobic interaction and hydrogen bond, Ligplot was used (25). Secondary structure was evaluated by stride server (26). In order to determine salt bridge and visualization of our model, VMDv 1.9.2 was used (27). 

## Results and Discussions

Considering a docking based on HADDOCK, we constructed the structural model of an IR homodimer/insulin complex in the presence of insulin pdb file (code: 3INC) and homology modeling based on 3LOH and 3W11 PDBs. 

**Figure 2:**
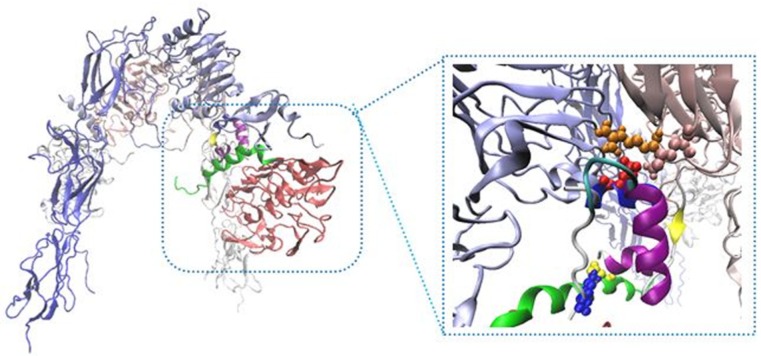
The interaction sites between insulin and IR. Receptor domains are shown in opaque

**Figure 3:**
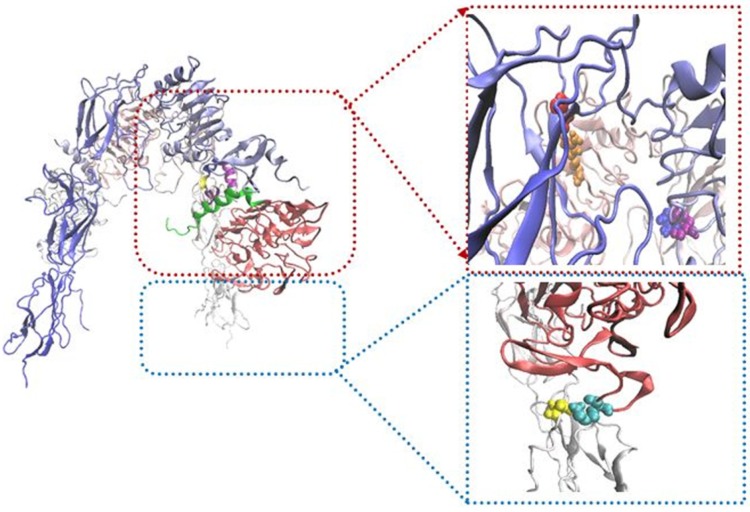
Schematic illustration showing location of salt bridges. In the right panels, salt bridges represent between R345 (L2; purple)-D535 (F1; blue), K166 (L1; cyan)-D645 (F2; yellow) and D499 (F1; red)-R371 (L2; orange

Our built model showed new salt bridges (i) Asp17 salt bridges to Arg554 (F1), the same residue salt bridges to Lys 484 (F1) (ii) the side chain of Lys50 salt bridges to Asp707 (α-CT) (Table 2 and Figure 2) (iii) the side chains of Asp499 (F1) interacted with Arg371 (L2) (iv) while the side chain of Arg345 (L2) interacted with Asp535 (F1) (v) the side chain of Lys166 (L1) interacted with Asp645 (F2) (Table 3 and Figure 3).

New Cartoons, and the A- and B-chain of insulin are secondary structure NewCartoons. New salt bridges between insulin and its receptor depicted as vdw (zoomed view). Blue vdw indicates K50 on insulin which interact with D707 (α-CT; yellow vdw), red vdw corresponds D17 on insulin had salt bridge with R554 (F1; orange vdw) and K484 (F1, pink vdw).

**Figure 4:**
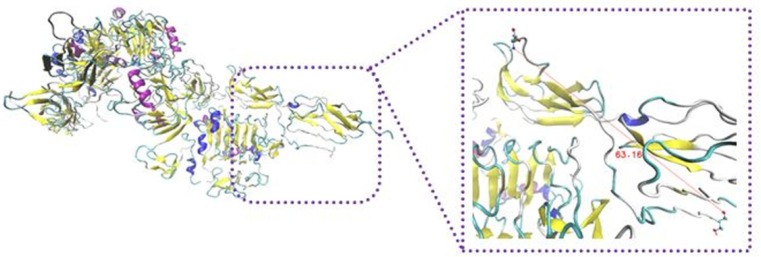
Schematic illustration of IR/insulin homodimer sequential docking approach. Distance between the mentioned regions were more than 50 Å, a restraint was defined in their connected hinge. It describes the power of salt bridges to expose rigid part 3 towards L1 on the opposite monomer

To survey the important role of salt bridges, rigid parts 2 and 3 resulting from HingeProt were added as two separate PDB files. To keep the distance between the mentioned rigid parts in the range of covalent bond, i.e., 1.4 ± 0.2, a restraint was defined in their connected hinge and three restraints defined for salt bridges between rigid part 3 and rigid part 1 (L1) of opposite monomer. Following docking, the distance between two rigid parts was in the range of 50 Å indicating the power of salt bridges to expose rigid part 3 towards L1 on the opposite monomer (Figure 4).

**Figure 5:**
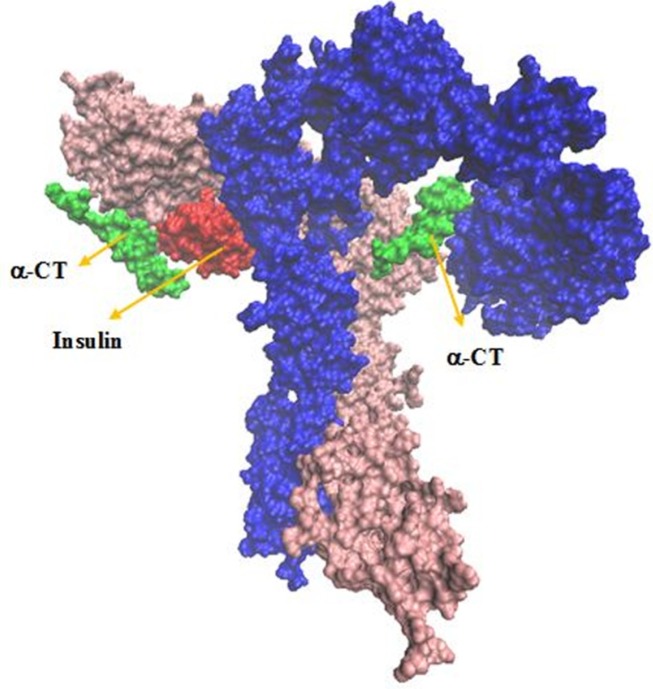
Schematic illustration of IR/insulin complex sequential docking method. Rigid parts 2 and 3 were added continually. The following docking, distance between these regions were about 4Å, but it displayed L shape instead of inverted V

To tackle this high distance, rigid part 2 and 3 were added as a complete PDB file. In spite of the distance between two rigid parts kept, there are not any conformational changes in the second structure of these rigid parts. To survey this event more accurately, the restraints that indicate salt bridge on rigid parts 2 and 3 were ignored. Then rigid parts 2 and 3 were added continually and docking was done in the same state. The new docking results display that their distance was about 4 Å, but instead of the formation of inverted V structure, L shape was occurred (Figure 5). 

These results indicated the importance of salt bridges to form an inverted V shape of IR. On the other hand, we suggest that the force which created space between rigid parts 2 and 3 during the application of salt bridges causes to pull rigid part 3 to extracellular regions and to transfer a force to intracellular parts; therefore these processes activate intracellular signaling pathways. 

With regard to the importance of α-CT, at first we focused on α-CT role in the complex of IR/insulin homodimer. Residue His710 contains a resonance structure and structural stability relative to the around residues, i.e., Asp707-Tyr708, 714, so His710 has the lowest affinity to create bond and also to participate in the interaction; the amount of affinity became too low especially in the presence of insulin.

**Figure 6:**
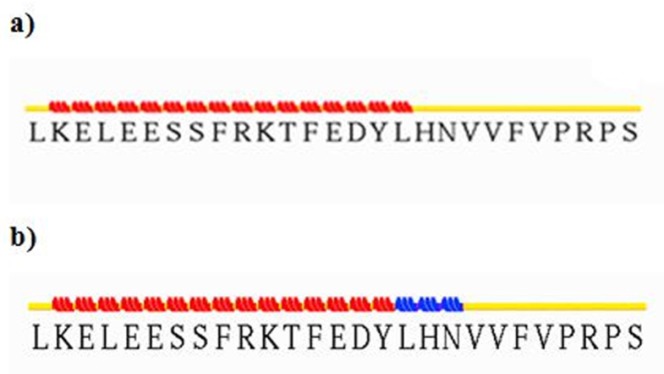
Input and output files of  -CT in complex with L1 resulting from stride server. In the input file, residue Leu709 contain  -helix structure, but residues His710 and Asn711 have turn shape. The following participation in the complex, residues 709-711 were converted to 3_10_-helix. It looks that the conformation gets extended relative to before docking

To elucidate secondary structure changes, stride server was used. In our results, turn and random coil were converted to extended beta sheet which took place in the most parts of the complex of insulin and IR dimer (Figure 6).

**Table 1:** Determination of hinge regions of insulin receptor by HingeProt. The combinations of both modes were used to build a complex of insulin and its receptor

Mode 1			
Rigid Part No	Residues	Score	
**1**	**E: 4-457**	**0.98**	
**2**	**E: 458-655, 693-710, 755-909**	**0.97**	
**Hinge residues: 457E**		
Mode 2			
Rigid Part No	Residues	Score	
**1**	**E: 4-254**	**0.98**	
**2**	**E: 255-598**	**0.96**	
**3**	**E: 599-655, 755-909**	**0.96**	
**4**	**E: 693-710**	**0.78**	
**Hinge residues: 254E, 598E, 655E, 710E**				

**Table 2:** List of generated salt bridges between insulin and rigid parts 2 &3. R554 & K484 (F1), D707 ( -CT) with residues 17 and 50 of insulin

Interaction sites	Distance (Å)
**D17-R554**	**2.68**
**D17-K484**	**2.64**
**K50-D707**	**2.62**

**Table 3:** List of salt bridges between rigid parts 2 & 3. D499 (F1), R345 (F1), K166 (L1), R371 & D535 (F1), D645 (F2).

Interaction sites	Distance (Å)
**D499-R371**	**2.73**
**R345-D535**	**2.59**
**K166-D645**	**2.66**

**Table 4:** Comparison of the experimental data between insulin and its receptor interaction sites with docking results of sequential method

Sequential docking methods						
Name	segment ID	experimental Active residue		number of interaction of HADDOCK predicted active site & experiment		the number of new predicted active site by HADDOCK
		Resid	number			
Rigid part 1 (1^th^ monomer)	A	32,34,36,37,39,65,62,64,88,89,94,96,120,124,153,154,247,246,248,254	20	17	85%	5
Rigid parts 2,3 (1^th^ monomer)	K	454,394,780,804,282,256,25,255	8	7	88%	34
Rigid part 4 (1^th^ monomer)	J	705,708,709,712,713	5	5	100%	8
Insulin	D	28,29,30,31,32,33,34,35,36,37,38,39,40,41,42,1,2,3,4,19,12,13,14,17	24	16	66%	11
Rigid part 1 (2^nd^ monomer)	I	247,246,248,254	4	4	100%	10
Rigid parts 2,3 (2^nd^ monomer)	E	484,552,591,602,616,620,62,780,804	9	6	66%	12

**Table 5:** The results of HADDOCK by using sequential docking method. Sequential docking method includes step 1: insulin & L1 domain (first monomer), step 2: output file of step1 & α-CT (second monomer), step 3: output file of step2 & FnIII-1 and FnIII-2 (second monomer), step 4: output file of step 3 & L1 domain (second monomer), step 5: output file of step 6 & α-CT (first monomer), step 6: output file of step 5 & FnIII-1 and FnIII-2 (first monomer).

Sequential docking method						
	Step 1	Step 2	Step 3	Step 4	Step 5	Step 6
HADDOCK score	-103.7+/-2.7	-119.8+/-1.9	-73.5+/-1.4	-57.0+/-2.2	-108.3+/-8.2	-231.6+/-7.0
Cluster size	64	129	198	200	191	200
RMSD*	0.8+/-0.5	0.4+/-0.2	0.4+/-0.3	0.3+/-0.2	0.6+/-0.5	0.5+/-0.3
VDW energy	-47.9+/-6.6	-75.8+/-5.2	-58.4+/-5.3	-30.8+/-1.1	-55.4+/-4.8	-114.4+/-12.7
Electrostatic energy	-142.4+/-25.5	-142.1+/-61.3	-323.2+/-20.0	-83.2+/-30.4	-200.0+/-29	-1003.7+/-12.1
Desolvation energy	-39.1+/-6.0	-36.1+/-11.1	1.2+/-4.0	-10.2+/-5.2	-24.7+/-10	74.2+/-14.9
Restraints violation energy	117.7+/-46.85	205.9+/-54.56	482.7+/-3.36	5.9+/-0.17	119.0+/-7.77	93.6+/-24.43
Buried Surface Area	1541.3+/-42.8	2028.6+/-62.6	2075.4+/-98.3	861.1+/-36.9	1652.0+/-59.3	5280.1+/-161
Z-Score	-1.3	-2.5	0.0	0.0	-1.0	0.0

* RMSD from the overall lowest-energy structure.

**Figure 7:**
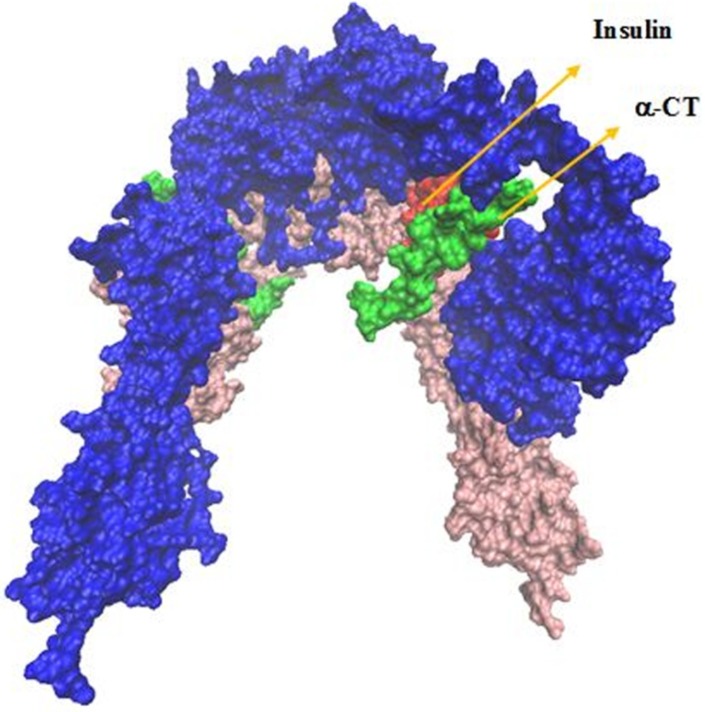
The IR ectodomain homodimer, showing the attached of insulin sequential docking approach. Red arrows in the right part of figure indicate viewing directions for insulin and CT peptide

**Figure 8:**
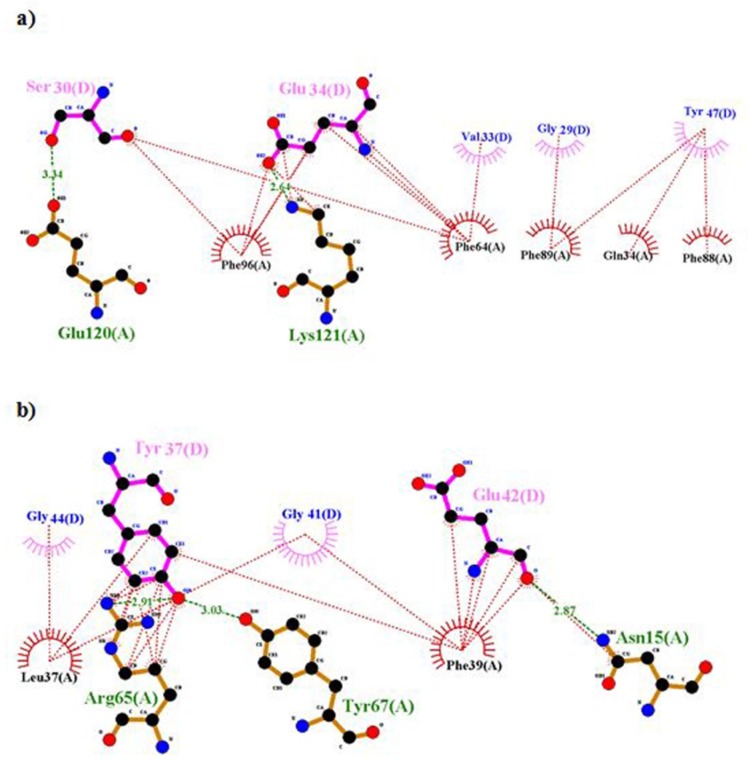
Ligplot representation of L1 and insulin. Output file of Ligplot represents hydrophobic interactions and hydrogen bonds between residues of rigid part 1 such as 34, 37, 39, 64, 65, 96, 88 and 89 with insulin; therefore they can’t interact with  -CT

**Figure 9.**
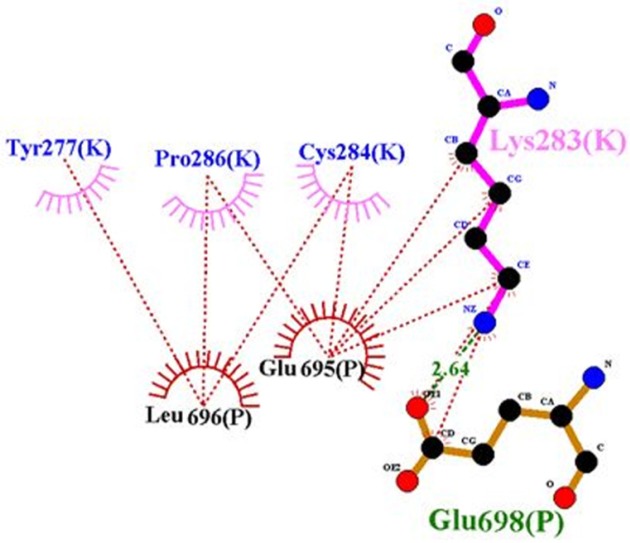
Ligplot representation of rigid part 3 of the first monomer and rigid part 3 of the second monomer. Residues 283, 4 and 286 of the first monomer interact with rigid part 3 of the second monomer; hence they prohibit the presence of residue 282 in the suitable interaction

**Figure 10:**
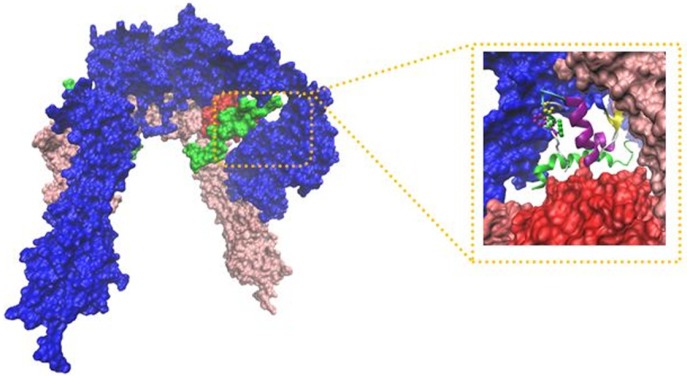
Representation of IR/insulin complex. Three residues of insulin, i.e., 44, 45, and 46 lie in the outside surface of L1,  -CT and CR. They don’t hold into the volume between the  CT segment, the L1– 2 surface and the adjacent CR domain

The extended beta sheet of secondary structures of insulin/IR complex is due to the formation of hydrogen bonds between protein atoms. Intracellular hydrogen bonds lead to be more stability in the protein structure (28). Significant hydrogen bonds were created between different regions of IR and insulin. Rigid parts 2 and 3 domains of the second monomer have the major contribution in the hydrogen bond and hydrophobic interactions. Because of closing rigid part 3 to L1 domain of the first monomer and generation hydrophobic interaction and salt bridges between them, both regions contain notable hydrogen and hydrophobic interactions. Our data indicated that the secondary structure of protein became looser than turn and coil shape. 

To survey the conformational change in residues Asn711-Val715 of α-CT, docking results were analyzed by Ligplot and stride server. Because of residues 711-715 are hydrophobic amino acids such as Val, Phe, and Asn, so when these residues interacted with hydrophilic residues of insulin like Tyr, Arg, Glu, and His, it causes to lie α-CT in a suitable position relative to insulin and they interact each other appropriately.

To evaluate the accuracy of constructed model, the resulted structure was compared with active sites obtained from Lawrence’s articles (8, 12) (Table 4). Comparing Tables 4 with experimental results show that sequential docking method is a suitable approach to survey the interaction between two molecules because we applied additional information during the docking. This information included the order of components participation in docking processes to create a complex of insulin/IR dimer.

Our analysis in sequential docking showed input active sites of rigid part 4 of the first monomer participated in the interaction completely. Rigid part 4 of the second monomer interacted with insulin; rigid parts 1, 2 and 3 of the first monomer were compatible more than 80% with input data. Insulin was consistent with experimental data more than 60%. Illustration schematic of insulin/IR dimer by using sequential method has been represented in Figure 7. 

The origin of the difference between our model and experimental data are discussed in the following:

According to Lawrence’s articles (8), hydrophobic face of α-CT comprising Phe705, Tyr708, Leu709, Val712 and Val713 engages a non-polar groove on L1β2 formed by Leu36, Leu37, Leu62, Phe64, Phe88, Phe89, Val94, and Phe96 (8). But in our model, some of them, i.e., residues 36, 62 and 94 were not involved in the interaction. There are hydrophobic interactions and hydrogen bonds between residues of rigid part 1 such as 34, 37, 39, 65, 64, 96, 89, and 88 with insulin, so these residues have an affinity for insulin and they cannot interact with -CT (Figure 8). But when residues of insulin, i.e., 30, 33, 34, 37, and 38 created hydrophobic interaction and hydrogen bond with rigid part 1, these residues prevented their neighbors, i.e., 31, 32, 35, 36, 39, and 40 to interact with rigid part 1. The same events happened for residue Asn282 in rigid part 3 of the first monomer. Residue 282 interacted with L1 of the first monomer (8), but in our model, hydrophobic interaction and hydrogen bond between its around residues, i.e., 284, 283, and 286 with rigid part 3 of the second monomer prohibited the presence of residue 282 in the suitable interaction (Figure 9).

In the experimental studies, situation and structural change of residues Gly44, Phe45, and Phe46 are unsolved (8), but our results showed the interaction between residues 44-37, 45-14, 45-37, and 46-14, it makes to lie the mentioned residues of insulin in the outside surface of L1-β2, CR and α-CT (Figure 10).

All output files resulting from HADDOCK have been shown in Table 5. According to the results of this table, the number of non-polar (hydrophobic) and charge amino acids interacted with each other in the step 4 are more than its previous and next phases in the sequential docking method. So this step has the most electrostatic and VDW energy and also the lowest buried surface area relative to the whole processes. Since step 4 of sequential docking method has the most negative VDW and electrostatic energy, so we can conclude the interaction sites of this stage is stronger than other phases. Based on this result, previous steps involve in the initiation of interaction between insulin and its receptor, but step 4 leads to make a tight contact due to the highest electrostatic and VDW energy.

## Conclusions

We constructed a complex of insulin receptor dimer and insulin based on sequential docking method for which HADDOCK is used as docking platform. We present evidence about a significant role of three salt bridges being involved in the formation of a complex of insulin and its receptor. As a consequence, the role of salt bridges is actually necessary to form an insulin/ IR complex and an inverted V structure to initiate intracellular signaling cascades. In addition to presence salt bridges, α-CT plays an importance role to interact with insulin and also new insulin/IR contacts, particularly at site 2 were foretokened to lie α-CT in a suitable situation relative to insulin and these residues interact with each other, appropriately. These results have been made in the elucidation of the structure-function relationship of insulin and insulin receptor binding, as well as providing a suitable model of the ligand-receptor complex and valuable information which can be applied to design several drugs. Since this part of IR plays a vital role to bind to insulin, so novel anti-diabetic peptides and even small molecules can be designed to improve the affinity binding of α-CT relative to insulin.
